# Giant viruses coexisted with the cellular ancestors and represent a distinct supergroup along with superkingdoms Archaea, Bacteria and Eukarya

**DOI:** 10.1186/1471-2148-12-156

**Published:** 2012-08-24

**Authors:** Arshan Nasir, Kyung Mo Kim, Gustavo Caetano-Anolles

**Affiliations:** 1 Evolutionary Bioinformatics Laboratory, Department of Crop Science, University of Illinois, Urbana, IL, 61801, USA; 2 Korean Bioinformation Center, Korea Research Institute of Bioscience and Biotechnology, 111 Gwahangno, Yuseong-gu, Daejeon, 305-806, Korea

## Abstract

**Background:**

The discovery of giant viruses with genome and physical size comparable to cellular organisms, remnants of protein translation machinery and virus-specific parasites (virophages) have raised intriguing questions about their origin. Evidence advocates for their inclusion into global phylogenomic studies and their consideration as a distinct and ancient form of life.

**Results:**

Here we reconstruct phylogenies describing the evolution of proteomes and protein domain structures of cellular organisms and double-stranded DNA viruses with medium-to-very-large proteomes (giant viruses). Trees of proteomes define viruses as a ‘fourth supergroup’ along with superkingdoms Archaea, Bacteria, and Eukarya. Trees of domains indicate they have evolved via massive and primordial reductive evolutionary processes. The distribution of domain structures suggests giant viruses harbor a significant number of protein domains including those with no cellular representation. The genomic and structural diversity embedded in the viral proteomes is comparable to the cellular proteomes of organisms with parasitic lifestyles. Since viral domains are widespread among cellular species, we propose that viruses mediate gene transfer between cells and crucially enhance biodiversity.

**Conclusions:**

Results call for a change in the way viruses are perceived. They likely represent a distinct form of life that either predated or coexisted with the last universal common ancestor (LUCA) and constitute a very crucial part of our planet’s biosphere.

## Background

The last few years have seen a dramatic increase in our knowledge of viruses particularly boosted by the discovery of giant viruses such as *Acanthameoba polyphagamimivirus*[1], *Acanthamoeba castellaniimamavirus*[2], and *Megavirus chilensis* (Megavirus) [3]. Mimivirus is truly a ‘Gulliver among the Lilliputians’ [4] as its sheer physical (~750 nm in diameter) and genomic (1.2 Mb, 1,018 genes) size exceeds those of the vast majority of viruses and dozens of cellular species, including numerous parasitic bacteria [5-8]. Mamavirus, an even bigger relative of mimivirus, was isolated from a cooling tower in Paris, and found associated with a new type of satellite virus, ‘Sputnik’ [2]. Sputnik appears to be a virophage, a parasite very much alike those of cellular organisms [9]. Both mimiviruses and mamaviruses have been identified as a new family (mimiviridae) of an apparently large monophyletic group known as the ‘Nucleocytoplasmic Large DNA viruses’ (NCLDV), which already includes the *Poxviridae*, *Irido/Ascoviridae*, *Phycodnaviridae*, *Marseilleviridae*, and *Asfarviridae*[10,11]. Megavirus was isolated from Chile and is the largest virus known to date (1.26 Mb, 1,120 genes) [3]. Both mimiviruses and megaviruses encode for genes that were never encountered in viral genomes before, such as genes related to DNA repair, protein folding and most surprisingly protein translation [3,5]. Some other fascinating features include the unique ‘Stargate’ DNA entry-exit mechanism [12] and the presence of highly conserved early promoter elements [3,13]. These features make giant viruses the most complex viruses known to date, reviving debates about their origin and evolution [4,7,11,14-18]. Some scientists believe mimiviruses are representatives of a distinct supergroup that is united by the presence of capsid (i.e., protein structures that enclose viral genetic material and are found in many diverse viruses) [15], and should be placed in the tree of life (ToL) along with superkingdoms, Archaea, Bacteria and Eukarya [19,20]. They propose that mimiviruses (and megaviruses) evolved from a more complex ancestral virus by means of reductive evolution (i.e., by the progressive loss of an evolving gene set) [3]. Others believe that the unusual set of mimiviral genes was acquired from its ameobal host by horizontal gene transfer (HGT) [18,21,22].

In this study, we take advantage of molecular processes occurring at higher and conserved levels of the structural hierarchy [23] and make statements about the evolution of cellular organisms and viruses that have large genomes. These molecular processes are responsible for the redundant appearance and accumulation of modules in the structure of living organisms [e.g., how many times particular protein domains are present in proteomes (i.e., genomic abundance)] [24]. The structural hierarchy defined in the Structural Classification of Proteins (SCOP) groups protein domains with high sequence conservation (>30% identities) into fold families (FFs), FFs with structural and functional evidence of common origin into fold superfamilies (FSFs), FSFs with common topologies (i.e., same major secondary structure in same arrangement) into folds (Fs) and Fs with similar secondary structure (e.g., alpha helix, beta sheet etc.) into protein classes [25,26]. A total of 110,800 domains that are indexed in SCOP ver. 1.75 correspond to 38,221 protein data bank (PDB) entries and are grouped into 1,195 Fs, 1,962 FSFs and 3,902 FFs. In contrast, the number of protein sequence entries in UniProtKB/SwissProt is more than half a million (535,698 entries as of 04/18/2012). The relatively limited number of structural designs suggests that structure space is finite and evolutionarily more conserved than sequence space [23,27]. Because domains defined at higher levels of SCOP classification (i.e., FSFs) exhibit higher levels of evolutionary conservation than domains defined at the lower fold family (FF) or sequence levels, they make useful tools (phylogenetic characters) when studying the evolution of protein domains and organisms [28,29]. This focus on structure as general evolutionary principle of biology offers several advantages over standard phylogenetic methods and overcomes important limitations imposed by the violation of assumptions that occur when attempting to extract deep phylogenetic signal present in molecular sequence data [24]. For example, phylogenomic trees derived from genomic abundance counts of FF and FSF domains [28,30] are less prone to the effects of HGT as shown in a number of studies [29,31-34] and do not require computation of sequence alignment, making them free from the problems resulting from characters that are not applicable to data sets (i.e., insertion/deletions in sequence alignment) [35,36]. Trees also sample hundreds of proteomes and the set of entire FFs and FSFs. Thus, taxa are finite and insensitive to taxon sampling problems. More importantly, tree reconstruction is free from the violations of character independence that plague sequence analysis because of the mere existence of molecular structure [37]. The phylogenomic analysis of structure has been used previously to reconstruct ToLs and reveal the early divergence of Archaea relative to Bacteria and Eukarya [28-30], as well as reductive trends in the proteomes of cellular organisms [28,38]. The structural make up of the last universal common ancestor (LUCA) of life was also reconstructed [29]. Molecules and planetary events (inferred from geology and paleobiology) were linked with a clock of folds [39]. Phylogenomic methods also uncovered the origin and history of ribosomes [40], among many other studies [41-46]. Here we make evolutionary statements from a census of abundance of 1,830 FSFs (defined in SCOP ver. 1.75) in a total of 1,037 proteomes. This *total* dataset included completely sequenced proteomes from 652 Bacteria, 259 Eukarya, 70 Archaea and 56 viruses. For the viral supergroup, we sampled double-stranded DNA (dsDNA) viruses with medium-to-very large proteomes (mainly NCLDV) since they harbor large genomes (i.e., giant viruses) and apportion maximum structural and genetic diversity to the virosphere (i.e., the group of all viruses) [47]. We test whether giant viruses follow the same patterns of reductive evolution we have seen in the cellular proteomes [28,38]. We also explore if they mediate transfer of domains between cells, enhancing planetary biodiversity and acting as source of new fold structures [47]. Finally, we place sampled viruses in a *universal tree of life* (uToL) and propose they embody a new and ancient supergroup that either predated or coexisted with LUCA. This supergroup experienced massive gene loss very early in evolution resulting in a transition to the parasitic lifestyle.

## Methods

### Data retrieval

We downloaded the FSF assignments for a total of 981 organisms with publically available sequenced genomes (70 Archaea, 652 Bacteria, and 259 Eukarya) from the SUPERFAMILY ver. 1.75 MySQL database (release: 08/29/2010)[48,49]. We retrieved the protein sequences encoded by 56 viral genomes including 51 NCLDV and 5 viruses from Archaea, Bacteria and Eukarya (united by the presence of capsid) from the NCBI viral genome resource homepage (link: http://www.ncbi.nlm.nih.gov/genomes/GenomesHome.cgi?taxid=10239) and assigned structural domains corresponding to 1,830 FSFs using the hidden Markov Models (HMMs) of structural recognition in SUPERFAMILY at a probability cutoff *E* value of 0.0001 [50]. This defined a *total* dataset of 1,037 proteomes (56 viruses, 70 Archaea, 652 Bacteria, and 259 Eukarya) with a total FSF repertoire of 1,739 FSFs (91 out of 1,830 FSFs had no representation in our dataset and were excluded from the analysis). In these studies, domains were identified using *concise classification strings* (css) (e.g., c.26.1.5, where c represents the protein class, 26 the F, 1 the FSF and 5 the FF).

### Phylogenomic analysis

We generated rooted phylogenomic trees describing the evolution of protein domains (ToDs) and proteomes (ToPs) using the genomic abundance counts of FSFs as phylogenomic characters [41,43]. We began by counting the number of times each FSF was present in every proteome for the *total* dataset. We defined this count as the genomic abundance value (*g*) and presented these values in a 1,037 * 1,739 ([total number of proteomes] × [total number of FSFs]) matrix. Because large genomes (e.g., *Homo sapiens*) are expected to have higher counts of FSFs, *g* values can range from 0 (absent) to thousands [41,51]. In order to account for unequal genome sizes and large variances, and because most phylogenetic software allow characters states only up to 32, we normalized the *g* values to a 0–23 scale in an alphanumeric format (0–9 and A-N) using the following formula [41,51].

*g*_*ab_norm*_ = Round [ln(*g*_*ab*_ + 1)/In (*g*_*max*_ + 1) * 23 ]

Where, *a* and *b* represent an FSF and a proteome respectively; *g*_*ab*_ describes the *g* value of the FSF *a* in the proteome *b; g*_*max*_ is the maximum *g* value in the matrix; Round function normalizes the *g*_*ab*_ value taking into account the *g*_*max*_ and standardizes the values to a 0–23 scale (*g*_*ab_norm*_) [41,51]. The normalization results in 24 transformed values that are compatible with PAUP* ver. 4.0b10 [52] and represents linearly ordered multistate phylogenetic character states.

For the ToDs, we declared N (maximum value) as the ancestral character state under the assumption that the most abundant FSF (character N) appeared first in evolution. We expect older FSFs to be abundant as gene duplications and rearrangements increase proteomic repertoires. Under this scenario, older FSFs have more time to increase their representations and are ‘reused’ as modules for the generation of new domain architectures [30,46]. For this reconstruction, FSFs were treated as taxa and proteomes as characters. For the ToPs, we declared 0 (minimum value) as the ancestral character state assuming that the primordial proteome had a simpler organization and there was a progressive trend towards organismal complexity. Under this model, we assume that proteomic growth is governed by the accumulation of new domains and using old architectures in various combinations to enhance proteomic diversity [30,46]. In order to improve ToP reconstructions, we manually studied the lifestyles of cellular organisms in the *total* dataset and excluded organisms exhibiting parasitic (P) and obligate parasitic (OP) lifestyles, as their inclusion is known to affect the topology of the phylogenetic tree [29]. We also sampled 50 proteomes equally from cells and dsDNA viruses to reduce any effect of the large number of bacterial proteomes in our dataset. For the reconstructions of ToPs, data matrix was transposed to represent proteomes as taxa and FSFs as characters. Ancestral states were declared using the ANCSTATE command and trees were rooted using the Lundberg method which does not require to specify the outgroup taxa but places the root at the most parsimonious location [53]. Maximum Parsimony (MP) was used to search for the best possible tree. MP is a meaningful way to analyze FSF data as we pool the set of entire FSFs into a single phylogenetic analysis. Therefore, our dataset encompasses many different genes that are changing with different evolutionary rates [54]. We note that MP gives superior performance when evolutionary rate is variable for the set of characters and approximates maximum likelihood (ML) when using large numbers of multistate characters (convergence is less likely with large number of character states) [54]. To evaluate the reliability of phylogenetic trees, we carried out a bootstrap analysis with 1,000 replicates. From the ToDs, we calculated the relative age of each FSF defined as the node distance (*nd*) using a PERL script that counts the number of nodes from a hypothetical ancestral FSF at the base of the tree to each leaf and provides it in a relative 0–1 scale. In order to evaluate homoplasy (i.e., conflict between data and tree) affecting the ToPs, retention indexes (*r*_*i*_) were calculated for individual FSF characters using the ‘DIAG’ option in PAUP* [55]. *r*_*i*_ portrays processes other than vertical inheritance (i.e., convergent evolution and HGT) of characters (FSFs) on a relative scale of 0–1 and is insensitive to the large number of taxa [55]. Higher *r*_*i*_ values indicate a better fit of characters (FSFs) to the phylogeny and support vertical inheritance. Trees were visualized using Dendroscope ver. 2.4 [56].

### Reconstruction of network trees

We generated network trees for 200 proteomes sampled equally from Archaea, Bacteria, Eukarya and the giant viruses. Network trees were constructed using the NeighborNet algorithm [57] implemented in the SplitsTree4 package [58]. The presence/absence matrix for a total of 1,739 FSFs was used as characters to describe the 200 taxa (the abundance matrix was incompatible with the software). Thus taxa were treated as binary sequences where 1 and 0 represented presence and absence of FSFs, respectively. SplitsTree4 evaluates each non-constant column in the matrix and creates a split of the taxon set. For example, if an FSF is present only in Archaea and Bacteria, but absent from Eukarya and viruses then the taxa will be partitioned into two sets i.e., (Archaea, Bacteria) and (Eukarya, viruses). The algorithm operates by first generating a distance matrix that is used to infer the set of splits for the given taxa. The set of splits is visualized as a network that gives a generalized representation of the phylogeny. For this exercise, distances were computed using the UncorrectedP method that calculates the fraction of variable positions between any two taxa. The reliability of Splits was evaluated by 1,000 bootstrap replicates.

### Distribution of FSF domains in organismal groups

In order to evaluate the spread/popularity of FSFs across the three superkingdoms, Archaea (A), Bacteria (B) and Eukarya (E), and the viral supergroup (V) (which we collectively refer to as supergroups but reserve the word superkingdom for cellular supergroups), we assigned FSFs appearing in all supergroups to the ABEV category, those present in all but one supergroup to the ABE, AEV, BEV, and ABV categories, those present in two supergroups to the AB, AE, AV, BE, BV, and EV categories, and those present in only one supergroup to the A, B, E, and V categories (these individual categories will be referred to as taxonomic groups hereinafter). We used a distribution index (*f*) to describe the popularity of FSFs across all the proteomes. This index ranges from 0 to 1 and represents the fraction of proteomes harboring a particular FSF [28]. An *f* value of 1 indicates that a particular FSF is present in all the proteomes while a value close to 0 suggests it is present in very few proteomes or absent (i.e., probably lost from a lineage) [28].

### Estimating the bias in the spread of viral FSFs among cellular proteomes

The total repertoire of FSFs present in each superkingdom was split into two components: (i) the set of FSFs shared with the viral supergroup, and (ii) the set of FSFs of that superkingdom (shared or not shared with other superkingdoms). The *f* index for both components was represented in a boxplot generated using programming implementations in R ver. 2.10.1.

### Enrichment of taxonomic groups with viral FSFs

In order to estimate which taxonomic groups were enriched with viral FSFs, we compared the counts of FSFs in cellular taxonomic groups (background) against the viral taxonomic groups (sample). The probability of enrichment of a particular taxonomic group was calculated using the hypergeometric distribution and the equation [29,32]:

(1) $$P\left(X=k\right)=\frac{\left(\begin{array}{l} M\\ k \end{array}\right)\left(\begin{array}{l} N-M\\ n-k \end{array}\right)}{\left(\begin{array}{l} N\\ n \end{array}\right)},where\;\left(\begin{array}{l} a\\ b \end{array}\right)=\frac{a!}{b!\left(a-b\right)!}$$

Where, *M* indicates the number of FSFs in the background; *k* indicates the number of FSFs in the sample; *N* is the total number of FSFs of the two sets; *n* represents the total number of FSFs in sample; and *P(X = k)* is the probability that implies a chance that a random variable *X* has *k* FSFs for a given taxonomic group [29]. Referring to the equation above and previous literature [29,32], we calculated *P* values for the individual sub-categories that had *k/n* larger (over-represented) or smaller (under-represented) than *M/N* and evaluated statistical significance with 95% confidence level (*P* < 0.05).

### Functional annotation of viral FSFs

We used the functional annotation scheme described by Vogel and Chothia to assign molecular functions to viral FSFs [59-61]. This annotation is based on one-to-one mapping between FSFs and molecular functions and utilized before to describe the functional make up of cellular proteomes [27]. Vogel divides molecular functions into 7 major categories including, *Metabolism*, *Information*, *Intracellular processes (ICP)*, *Extracellular processes (ECP)*, *Regulation*, *General* and *Other*. These major categories are further subdivided into 50 minor categories (Table 1). The classification scheme is based upon manual surveys and various online resources like Cluster of Orthologus Groups (COGs) and Gene Ontology (GO) databases [62,63]. For multifunctional FSFs, the most dominant function is assigned to the FSF, while the error rate is assessed to be <10% for large FSFs and <20% for all FSFs [59-61]. 

**Table 1:** **Mapping between the major and minor functional categories described in**[59-61]

**Major category**	**Minor categories**
*Metabolism*	Energy, Photosynthesis, E-transfer, Amino acids m/tr, Nitrogen m/tr, Nucleotide m/tr, Carbohydrate m/tr, Polysaccharide m/tr, Storage, Coenzyme m/tr, Lipid m/tr, Cell envelope m/tr, Secondary metabolism, Redox, Transferases, Other enzymes
*Information*	Chromatin structure, Translation, Transcription, DNA replication/repair, RNA processing, Nuclear structure
*Intracellular processes*	Cell cycle, Apoptosis, Phospholipid m/tr, Cell motility, Trafficking/secretion, Protein modification, Proteases, Ion m/tr, Transport
*Regulation*	RNA binding, m/tr, DNA-binding, Kinases/phosphatases, Signal transduction, Other regulatory function, Receptor activity
*General*	Small molecule binding, Ion binding, Lipid/membrane binding, Ligand binding, General, Protein interaction, Structural protein
*Other*	Unknown functions, viral proteins
*Extracellular processes*	Cell adhesion, Immune response, Blood clotting, Toxins/defense

m/tr, metabolism and transport.

## Results

### The overlap between mimiviruses and parasitic microbes is significant

The HMMs detected a large number of domains (4,679) and a rich repertoire of FSFs (304 distinct FSFs) in the medium-to-very-large viral proteomes we sampled (Table 2). Six out of these FSFs were viral-specific and had no representation in the cellular proteomes (Table 3). These viral-specific FSFs are responsible for functions unique to viruses, such as attachment to the host cell receptors and DNA, inhibition of caspases to trigger anti-apoptosis, and acting as major capsid proteins (Table 3). The median proteomic coverage (i.e., proteins with FSF assignments / total number of proteins) in viral proteomes was 34%, while *A. polyphaga* mimivirus had the highest coverage (59%) (Table 2). A comparison with the cellular supergroups (Additional file 1: Table S1) revealed that the proteomic coverage of mimivirus was higher than the median coverage in eukaryotes (55%) but lower compared to the bacterial (66%) and archaeal (61%) proteomes. The range of assignments in cells varies from 22-80% in Eukarya, 44-88% in Bacteria and 52-71% in Archaea (Additional file 1: Table S1). We note that mimiviruses overlap with cellular species that are similar in genome size and lifestyles. For example, despite considerable proteomic coverage, mimiviral assignments were restricted to only 163 distinct FSFs, a rather poor repertoire when compared for example with FSFs present in the proteomes of free living (FL) organisms (ranging from 407 FSFs in *Staphylothermus marinus* to 1,084 in *Capitella* sp.). FSF number was however comparable to small organisms with reduced genomes or parasitic/symbiotic lifestyles [e.g., *Guillardia theta* (189 distinct FSFs), *Nanoarchaeum equitans* (211 FSFs) and *Candidatus* Hodgkinia cicadicola (115 FSFs)]. The average reuse level of mimiviral FSFs (total FSFs/distinct FSFs) was quite low as well (530/163 = 3.25) but still comparable to that of organisms with similar genome size or lifestyles (e.g., 3.03 in *Staphylothermus marinus*, 1.42 in *Candidatus* Hodgkinia cicadicola, 2.01 in *Nanoarchaeam equitans,* and 2.48 in *Guillardia theta*). In summary, a significant number of FSFs exist in the proteomes of dsDNA viruses, including the ones that are not encoded by cells. Mimiviruses have a genome size comparable to numerous small bacteria and also share with them a very simple proteome. Both features overlap significantly with the parasitic unicellular organisms. 

**Table 2:** **List of dsDNA viruses sampled along with family name, total number of proteins, number of total and unique FSFs detected by the SUPERFAMILY HMMs (*E*-value cutoff of 0.0001) and proteomic coverage**

**#**	**Common name**	**Virus family**	**No. of proteins**	**Total FSFs detected**	**Total distinct FSFs**	**Proteomic coverage (%)**
1	Human Adenovirus	Adenoviridae	36	15	11	42
2	Heliothis virescens ascovirus 3e	Ascoviridae	180	39	24	22
3	Spodoptera frugiperda ascovirus 1a	Ascoviridae	123	36	22	30
4	Trichoplusia ni ascovirus 2c	Ascoviridae	164	49	29	30
5	African swine fever virus	Asfarviridae	160	55	37	35
6	Pseudoalteromonas phage PM2	Corticoviridae	22	1	1	5
7	Ambystoma tigrinum virus	Iridoviridae	95	33	23	35
8	Singapore grouper iridovirus	Iridoviridae	162	48	31	30
9	Infectious spleen and kidney necrosis virus	Iridoviridae	125	40	26	32
10	Lymphocystis disease virus - isolate China	Iridoviridae	239	47	30	20
11	Aedes taeniorhynchus iridescent virus	Iridoviridae	126	54	35	43
12	Frog virus 3	Iridoviridae	99	30	22	31
13	Invertebrate iridescent virus 6	Iridoviridae	468	77	48	17
14	Lymphocystis disease virus 1	Iridoviridae	110	36	26	33
15	Soft-shelled turtle iridovirus	Iridoviridae	105	31	22	30
16	Acanthamoeba polyphaga mimivirus	Mimiviridae	911	530	163	59
17	Marseillevirus	Marseilleviridae	856	264	61	31
18	Acanthocystis turfacea Chlorella virus 1	Phycodnaviridae	860	158	78	19
19	Emiliania huxleyi virus 86	Phycodnaviridae	472	105	58	23
20	Feldmannia species virus	Phycodnaviridae	150	55	33	37
21	Paramecium bursaria Chlorella virus 1	Phycodnaviridae	689	155	72	23
22	Paramecium bursaria Chlorella virus AR158	Phycodnaviridae	814	184	76	23
23	Paramecium bursaria Chlorella virus FR483	Phycodnaviridae	849	147	71	18
24	Paramecium bursaria Chlorella virus NY2A	Phycodnaviridae	886	209	74	24
25	Ectocarpus siliculosus virus 1	Phycodnaviridae	240	97	42	41
26	Ostreococcus tauri virus 1	Phycodnaviridae	230	104	56	46
27	Ostreococcus virus OsV5	Phycodnaviridae	264	100	58	38
28	Bovine papular stomatitis virus	Poxviridae	131	42	24	33
29	Amsacta moorei entomopoxvirus 'L'	Poxviridae	294	66	37	23
30	Camelpox virus	Poxviridae	211	93	45	45
31	Canarypox virus	Poxviridae	328	157	50	48
32	Cowpox virus	Poxviridae	233	113	47	49
33	Crocodilepox virus	Poxviridae	173	47	26	28
34	Deerpox virus W-1170-84	Poxviridae	170	73	39	43
35	Ectromelia virus	Poxviridae	173	91	45	53
36	Fowlpox virus	Poxviridae	261	118	44	46
37	Goatpox virus Pellor	Poxviridae	150	66	36	44
38	Lumpy skin disease virus NI-2490	Poxviridae	156	68	36	44
39	Melanoplus sanguinipes entomopoxvirus	Poxviridae	267	83	37	32
40	Molluscum contagiosum virus subtype 1	Poxviridae	163	39	27	24
41	Monkeypox virus Zaire-96-I-16	Poxviridae	191	93	46	49
42	Myxoma virus	Poxviridae	170	78	40	46
43	Orf virus	Poxviridae	130	38	23	30
44	Pseudocowpox virus	Poxviridae	134	40	22	30
45	Rabbit fibroma virus	Poxviridae	165	79	40	48
46	Sheeppox virus 17077-99	Poxviridae	148	66	36	45
47	Swinepox virus	Poxviridae	150	64	39	43
48	Tanapox virus	Poxviridae	156	60	36	39
49	Taterapox virus	Poxviridae	225	98	45	44
50	Vaccinica virus	Poxviridae	223	98	46	44
51	Variola virus	Poxviridae	197	80	44	41
52	Yaba monkey tumor virus	Poxviridae	140	52	33	38
53	Yaba-like disease virus	Poxviridae	152	60	36	40
54	Sulfolobus turreted icosahedral virus	Rudiviridae	36	5	5	14
55	Bacillus phage Bam35c	Tectiviridae	32	6	6	19
56	Enterobacteria phage PRD1	Tectiviridae	31	7	6	23

**Table 3:** **Viral-specific FSFs and their molecular functions**^1^

**No.**	**SCOP ID**	**FSF*ccs***	**Description**	**Function(s)**
1	89428	b.126.1	Adsorption protein P2	Attachment of phage to host conjugative DNA complex
2	49894	b.28.1	Baculovirus p35 protein	Anti-apoptotic in infected insect cells by inhibiting caspases
3	49835	b.21.1	Virus attachment protein globular domain	Binding selectively to cell surface receptors
4	49749	b.121.2	Group II dsDNA viruses VP	Includes major capsid protein family
5	47724	a.54.1	Domain of early E2A DNA binding protein ADDBP	C-terminal domain of the viral DNA binding protein
6	57917	g.51.1	Zn binding domain of ADDBP	Binds to DNA and RNA & required for replication and transcription control.

^1^Abbreviations: ID, identifier; *ccs,* concise classification string.

### The distribution of domain structures is biased but shows widespread representation of viral FSFs

**Figure 1:**
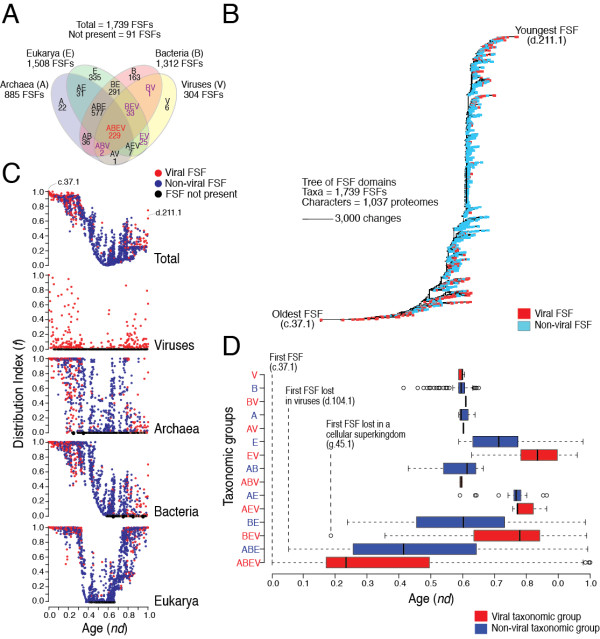
**History of protein domain structures.A**. The Venn diagram shows distribution of FSFs in the taxonomic groups. Viral families included in the analysis: Adenoviridae, Ascoviridae, Asfarviridae, Corticoviridae, Iridoviridae, Marseilleviridae, Mimiviridae, Phycodnaviridae, Poxviridae, Rudiviridae, and Tectiviridae (see Table 2). **B**. Phylogenomic tree of protein domain structure describing the evolution of 1,739 FSFs in 1,037 proteomes (4,63,915 steps; consistency index CI = 0.051; retention index RI = 0.795; tree skewness *g*_1_ = −0.127). Taxa are FSFs and characters are proteomes. Terminal leaves were not labeled, as they would not be legible. **C**. Distribution index (*f*, the number of species using an FSF/total number of species) of each FSF plotted against relative age (*nd*, number of nodes from the root/total number of nodes) for the four supergroups and individually for sampled viruses, Archaea, Bacteria, and Eukarya. **D**. Boxplots displaying distribution of FSFs in viral and cellular taxonomic groups with respect to age (*nd*). Vertical lines within each distribution represent group median values. Dotted vertical lines represent important evolutionary events in the evolution of proteomes.

FSFs are not equally distributed in the proteomes of Archaea (A), Bacteria (B) and Eukarya (E), and viruses (V). In turn, FSFs exist that are uniquely present (groups A, B, E or V) or are shared by two (AB, AE, AV, BE, BV, EV), three (ABE, ABV, AEV, BEV) or all (ABEV). A Venn diagram (Figure 1A) describes the FSF distribution and highlights the differential enrichment of viral FSFs within these taxonomic groups. All cellular taxonomic groups share FSFs with the viral supergroup. ABE is the most populated with 557 FSFs, BE is the second largest with 291 FSFs and ABEV makes the third largest group with 229 FSFs. Eukaryotes have the highest number of supergroup-specific FSFs with 335 (~19%) of the total FSFs present only in eukaryotes, followed by Bacteria, Archaea and viruses with 163 (9.37%), 22 (1.26%) and 6 (0.345%) supergroup-specific FSFs, respectively. This complete and unique distribution of FSFs in supergroups suggests that viruses with medium-to-very-large proteomes maintain considerable structural diversity despite their reduced genomes and parasitic lifestyle. The lower number of viral-specific FSFs can be explained by the fact that current shift in genomics is towards the sequencing of viral genomes with medical importance [47]. The discovery and sequencing of viruses with large genomes (e.g., mimiviruses, megaviruses, and mamaviruses) is expected to add to the number of viral-specific structures. However, we expect that the relative patterns of FSF sharing with the cellular supergroups will remain conserved. 

### Reductive evolutionary processes explain viral make up

We generated trees of FSF domains from linearly ordered multistate phylogenetic features (FSFs as taxa and proteomes as characters) using maximum parsimony (MP) (Figure 1B). Trees of FSFs are rooted and highly unbalanced. However, the imbalance in trees results from the accumulation of protein domains in proteomes (i.e., genomic abundance) and portrays a biological process and not a phylogenetic artifact. From the FSF tree, we calculated the age of each FSF defined as the node distance (*nd*) [28]. *nd* is given on a relative 0–1 scale, with *nd* = 0 representing the origin of protein domains and *nd* = 1 the present [28]. *nd* is a good proxy for the age of each FSF, is linearly proportional to geological time, follows a molecular clock and can be used to accurately date domains defined at FF and FSF levels [39]. When plotted against the fraction of proteomes encoding each FSF (i.e., distribution index; *f*), *nd* described unprecedented patterns in the evolution and origins of proteomes in the *total* dataset and individual supergroups (Figure 1C).

We note that the majority of the viral FSFs originated either very early or very late, showing a clear bimodal pattern of domain appearance (red circles in the tree of Figure 1B and timelines of Figure 1C). The distribution of FSFs in the *total* dataset revealed that the most ancient FSF, the P-loop-containing NTP hydrolase (c.37.1) was present in all the proteomes (*f* = 1), including the viral proteomes (Figure 1C: Total). In total, 28 ancient FSFs had an *f* > 0.947 and were present in almost all cellular and most viral proteomes. However, the representation of FSFs decreased in the timeline with increasing *nd* until *f* approached 0 at about *nd* = 0.587 (Figure 1C:Total). The steady drop in the *f* value for ancestral FSFs (*nd* < 0.587) defines the reductive model of evolution for viral and microbial superkingdoms. We hypothesize that very early in the evolutionary timeline (*nd* < 0.587), *f* values smaller than 1 indicated loss of an existing FSF in few proteomes. In general, the probability for a few proteomes to loose an FSF was higher than the probability for the rest of the proteomes to discover the same FSF simultaneously (very much alike the probabilistic model for insertion and deletions in sequence alignment) [28]. This differential loss of structures probably triggered the early diversification of lineages emerging from an ancestral community (read below) [28]. The *f* value approached a minimum at *nd* = 0.587. Beyond this point, an opposite trend took place and the representation of FSFs in proteomes increased with increasing *nd*. We explain the increase in *f* value for younger FSFs (*nd* > 0.587) by evolutionary forces initiating the emergence of diversified supergroups (i.e., A, B, E, and V). These forces were primarily responsible for genome expansion in proteomes (especially in Eukarya) by evolutionary processes including gene shuffling, domain rearrangements, and HGT [28,46].

Distribution plots for the individual supergroups confirmed that, in general, the most ancient FSFs (*nd* = 0–0.4) were shared by most proteomes (Figures 1C). However, the representation of ancient FSFs decreased in time, first in the viral proteomes (Figure 1C: Viruses), and then in the cellular proteomes, starting with Archaea (Figure 1C: Archaea), then Bacteria (Figure 1C: Bacteria) and finally Eukarya (Figure 1C: Eukarya). The decrease in the representation of ancient FSFs (as explained above) is consistent with the reductive tendencies described previously for the cellular proteomes [28,38]. We propose that both sampled dsDNA viruses and Archaea experienced high levels of genome reduction through loss of ancient FSFs. While in general, they maintained small proteomic representations of younger FSFs, FSF representation increased considerably in the eukaryal proteomes as *f* reached 1 again at *nd* =1 (primarily triggered by domain rearrangements) (Figure 1C: Eukarya) [46].

### Appearance of supergroups

The distribution of FSFs with respect to age (*nd*) (Figure 1D) revealed that ABEV was the most ancient taxonomic group with *nd* ranging between 0–1 and a median *nd* of 0.2324. This confirmed that the majority of the FSFs shared between giant viruses and cells were ancient, providing further support to the hypothesis of early coexistence of giant viruses with cellular ancestors. The appearance of the ABEV taxonomic group was followed by the ABE, BEV, and BE taxonomic groups, in that order. The late appearance of supergroup-specific taxonomic groups suggests giant viruses and Archaea diversified much later and concurrently with Eukarya (*nd* = 0.5867) (Figure 1D). Interestingly, the appearance of BV, EV and AV FSFs occurs soon after the appearance of the respective supergroup-specific FSFs or after the diversification of the respective supergroups (i.e., B, E and A). We hypothesize that these FSFs were discovered when dsDNA viruses began to infect their hosts and adopted a parasitic lifestyle. This occurred when lineages of diversified cellular organisms were already in existence. Therefore, parasitic adaptation in the viral proteomes appears to be an afterthought most likely triggered by massive amounts of genome reduction experienced very early in evolution (*nd* < 0.587).

### Early reductive evolution of the translational machinery of giant viruses

**Figure 2:**
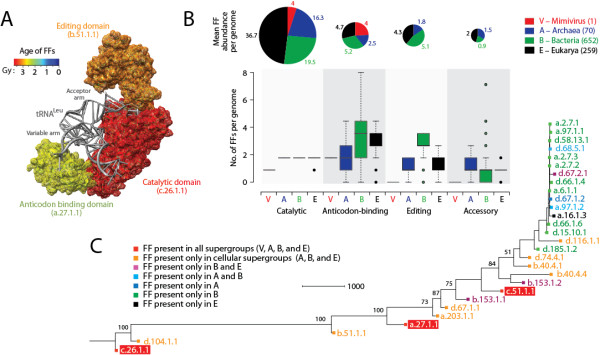
**Evolution of the major domains of aminoacyl-tRNA synthetase (aaRS) enzymes.A**. The leucyl-tRNA synthetase (LeuRS) enzyme in complex with tRNA^Leu^ (PDB entry 1WZ2) with its three domains (catalytic, editing and anticodon-binding) colored according to their age of origin. Domain ages were derived from a ToD at FF level of structural complexity [41]. Note how the variable arm of tRNA makes crucial contact with the anticodon-binding domain, which is evolutionarily derived, while the acceptor arm contacts the ancient catalytic domain in pre-editing conformation. **B**. Occurrence (box plots) and abundance (pie charts) of 28 fold family (FF) domains of aaRS enzymes with known structures in the total genomic dataset of 1,037 cellular organisms and viruses. The name and function of domains are described in Table S2 of the Additional file. **C**. Phylogenomic tree of protein domain structures describing the evolution of the 28 FFs of aaRSs in the 1,037 proteomes (982 parsimony informative characters; 26,638 steps; CI = 0.8479; RI = 0.8742; *g*_1_ = −0.1.401). Taxa are aaRS FF domains and characters are proteomes. FFs are labeled with SCOP concise classification strings. Numbers on the branches indicate bootstrap values. FF domains present in viruses are highlighted in red. Note that d.104.1.1 has been identified in megavirus (not included in this study).

The loss of FSFs was abrupt and massive for viruses. It started very early in evolution but substantially dropped in the *nd* = 0.4-0.6 range (Figure 1C: Viruses). Its first effects were on the repertoire of aminoacyl tRNA synthetase (aaRS) enzymes that are responsible for the algorithmic implementation of the genetic code [64-66]. The class II aaRS and biotin synthetase FSF (d.104.1) was the first domain structure to be completely lost in the viral proteomes we sampled (*f* = 0; *nd* = 0.0516) (Figure 1C: Viruses; boxplot for ABE in Figure 1D). This FSF includes the catalytic domain of class II aaRS enzymes that along with class I aaRS enzymes charge tRNAs with correct amino acids and make central components of the protein translation machinery [65,66]. It has been reported that the mimivirus genome encodes four class I aaRS enzymes (TyrRS, MetRS, ArgRS, and CysRS) but no class II enzymes [5]. Our census of FSFs confirms these findings. This partial enzymatic set of aaRSs was proposed to be a likely remnant of a primordial translational apparatus that was once present in the genome of its ancestor (virus or more likely cell) [3]. The recent discovery of megavirus (a distant phylogenetic relative of mimivirus that is not included in this study) led to the identification of seven aaRS in the megavirus genome including both the class I (TyrRS, MetRS, ArgRS, CysRS, TrpRS, and IleRS) and class II (AsnRS) enzymes [3]. Megavirus is therefore the only virus known to possess both class I and class II aaRSs. We studied the genomic distribution of 28 FF domains that make structural components of aaRS enzymes in the 1,037 proteomes of the *total* proteome dataset of cells and viruses (Figure 2). These structures are catalytic, editing, trans-editing, anticodon binding and accessory domains of aaRS enzymes (Additional file 2: Table S2). As we illustrate with LeuRS, each of these domains contribute their own history to the evolutionary make up of individual aaRS enzymes (Figure 2A). The vast majority of the FF domains were only present in cells. The viruses we sampled encoded only four instances of the catalytic domain of class I aaRS (c.26.1.1) and four total instances of the anticodon-binding domains of both class I (a.27.1.1) and class II aaRS (c.51.1.1) per proteome, all in mimivirus (Figure 2B). The fact that the anticodon-binding domain typical of class II ProRS, ThrRS, GlyRS and HisRS is present even if the correponding class II catalytic domain is absent is remarkable and suggests reductive evolutionary processes are still actively at play. The average number of catalytic domains per proteome was substantially larger in cells (ranging 16–37) and increased in the order Archaea, Bacteria and Eukarya (see pie charts of Figure 2B), following corresponding increases in genome complexity. The exception is the highly reduced eukaryotic genome of *Guillardia theta* that contains only one instance of an aaRS catalytic domain (corresponding to a class II enzyme) and resembles mimivirus. More importantly, we note that viruses did not contain editing or trans-editing domains that always appear together and at rather constant ratios with anticodon binding domains in cellular organisms and are especially substantial in Bacteria (bar diagrams, Figure 2B). Similarly, viruses did not contain any of the many other C-terminal and N-terminal accessory domains. These domains are typical of aaRSs and enhance their functional repertoire, especially in Eukarya [64]. As expected, a ToD reconstructed from the genomic abundance counts of these FFs (with FFs as taxa) described the origin and evolution of aaRS domains and revealed interesting patterns (Figure 2C). Catalytic and editing domains appeared at the base of the tree very early in evolution, generally before anticodon-binding domains necessary to establish crucial aminoacylation specificities. For example, the ValRS/IleRS/LeuRS editing domain FF (b.51.1.1) appeared before the anticodon binding domains that are present in viruses. Remarkably, the progression of loss of domains of aaRSs in supergroups (Figure 2C) follows the progression observed for the entire proteomic dataset (Figure 1). The reductive evolutionary tendencies of the aaRS enzymes are therefore not atypical. 

### uToLs identify viruses as a distinct supergroup along with cellular superkingdoms

**Figure 3:**
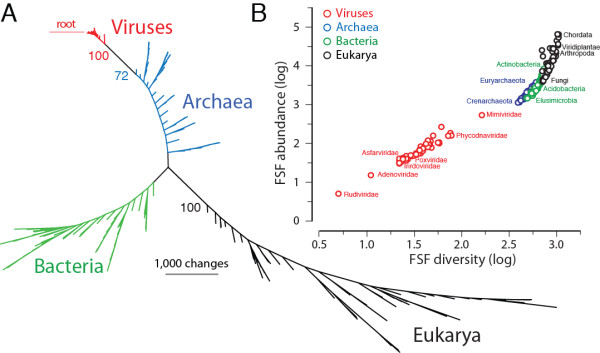
**Universal tree of life (uToL) and proteomic diversity.A**. One optimal most parsimonious phylogenomic tree describing the evolution of 200 proteomes (50 each from Archaea, Bacteria, and Eukarya and viruses; virus families are listed in Table 2) generated using the census of abundance of 1,739 FSFs (1,517 parsimoniously informative sites; 62,061 steps; CI = 0.156; RI = 0.804; *g*_1_ = −0.325). Terminal leaves of viruses (V), Archaea (A), Eukarya (E) and Bacteria (B) were labeled in red, blue, black and green respectively Numbers on the branches indicate bootstrap values. **B**. FSF diversity (number of distinct FSFs in a proteome) plotted against FSF abundance (total number of FSFs that are encoded) for 200 proteomes. Major families/phyla/kingdoms are labeled. Both axes are in logarithmic scale.

We reconstructed rooted uToLs built from the genomic abundance counts of individual FSFs (total character set: 1,739 FSFs) as previously described [28,29]. For this reconstruction, we excluded cellular organisms with P and OP lifestyles (in order to remove noise from the data) and sampled 50 proteomes equally from each supergroup [29]. The reconstruction produced trees in which organisms in Archaea, Bacteria, Eukarya, and viruses formed four distinct groups, placed viruses as the most ancient group and Archaea as the second oldest. Figure 3A gives a radial representation of an example phylogeny of randomly sampled taxa. The viral supergroup is discriminated from other superkingdoms by 72% bootstrap support. Both the viral and archaeal supergroups were always paraphyletic while Bacteria and Eukarya appeared monophyletic (Figure 3A). Reconstructions supported the early divergence of viruses and Archaea relative to Bacteria and Eukarya [28-30]. Because the FSF repertoire of sampled viruses (a total of 304 FSFs) is considerably smaller than the FSF repertoires in cellular supergroups (885 FSFs in Archaea; 1,312 FSFs in Bacteria; and 1,508 FSFs in Eukarya) (Figure 1A), we also reconstructed the uToL from the ABEV taxonomic group (Additional file 3: Figure S1). The ABEV taxonomic group includes 229 FSFs that are encoded by both sampled viruses and cells and is the most ancient group with a median *nd* of 0.2324 (Figure 1D). This exercise reduced the effect of the number of supergroup-specific structures in Archaea, Bacteria and Eukarya that are significantly greater than the viral-specific structures (22, 163, 335 VS 6) and eliminated any bias resulting from the phylogenomic model (i.e., we consider primordial proteomes to encode very few structures and root trees by structural absence). The uToL and network tree diagram (also read below) reconstructed from the set of 229 universal FSFs resulted in a topology which overall favored the previous reconstructions (Additional file 3: Figure S1). Viruses were identified as a separate group along with superkingdoms Archaea, Bacteria and Eukarya with the cellular world stemming from viruses and Archaea (Additional file 3: Figure S1). 

A plot that describes the interplay between diversity (use) and abundance (reuse) of FSFs (total number of distinct FSFs versus the total number of FSF domains that are encoded in a proteome) shows viruses have the simplest proteomes, followed progressively by Archaea, Bacteria and Eukarya, in that order (Figure 3B). Organisms follow a congruent trend towards structural diversity and organismal complexity. This trend confirms our initial evolutionary model of proteome growth that we use for the rooting of the uToL and again supports the ancestrality of viruses and Archaea [28].

### Phylogenomic networks give unity to sampled viruses

**Figure 4:**
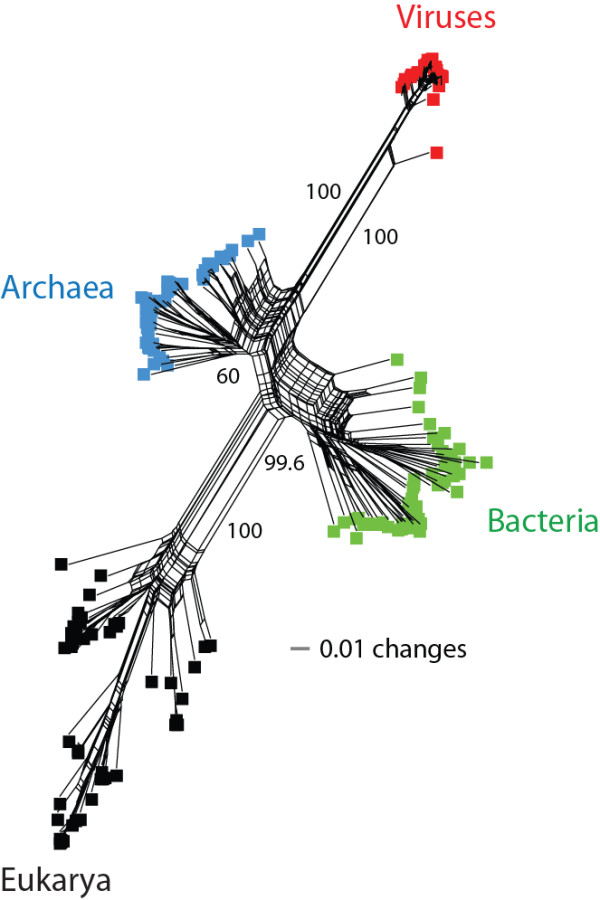
**Network tree visualization of the supergroups.** Network tree generated from the presence/absence matrix of 1,739 FSFs in 200 proteomes sampled equally from the four supergroups. The number of non-constant sites was 1,581. Nodes in the network tree are proteomes and are represented by rectangles labelled red, blue, green, and black for viruses, Archaea, Bacteria and Eukarya, respectively. Numbers on the major splits indicate bootstrap values.

When the evolutionary model involves processes like gene gain/loss, duplications, and HGT, it is appropriate to provide an abstract representation of the phylogeny using networks [57]. A phylogenomic network is expected to reflect the evolutionary tree when there is no conflict between data and the tree and aids in phylogenetic analysis. A network tree reconstructed by the agglomerative NeighborNet algorithm [57] identified viruses as a distinct supergroup along with cellular superkingdoms (Figure 4). Each edge on the network represented a split of taxa. The splits discriminating viruses and eukaryotes from the rest of the supergroups were supported by 100% bootstrap support. The network tree is congruent with the phylogeny recovered in Figure 3A and defies theories attributing large proteomes of dsDNA viruses to massive amounts of HGT from cells. In contrast, the resulting network gave unity to the sampled viruses and suggests vertical acquisition of their gene repertoires (no mixing of viruses with cells was observed in the network) (Figure 4). However, we realize that HGT from cells to viruses harboring smaller proteomes or RNA genomes might be occurring at different (or higher) levels. 

### Viruses enhance planetary biodiversity

**Figure 5:**
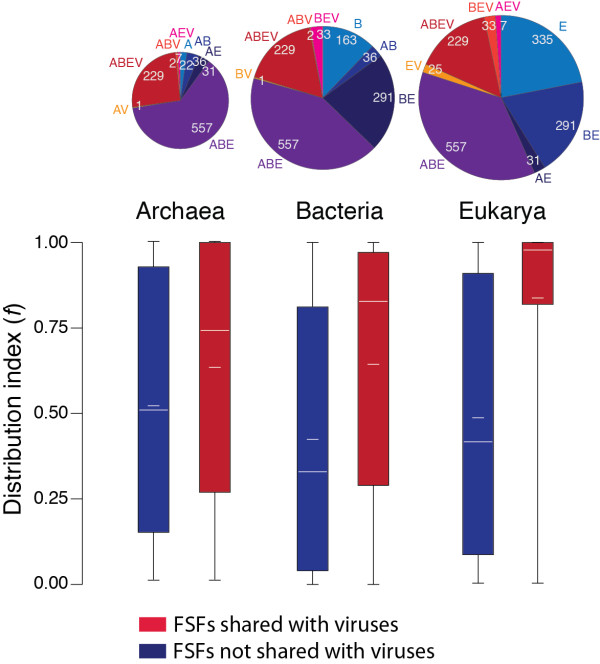
**Enrichment of viral FSFs.** Boxplots comparing the distribution index (*f*) of FSFs shared or not shared with viruses for each cellular superkingdom. Pie charts above each superkingdom represent distribution of FSFs in taxonomical groups within each superkingdom.

The spread (*f*) of viral FSFs relative to cellular FSFs in the individual proteomes of Archaea, Bacteria and Eukarya appeared considerably biased (Figure 5). When compared to the cell-specific FSFs, FSFs shared by viruses and cells were significantly widespread in the proteomes of a superkingdom. Viruses hold 294, 265, and 239 FSFs in common with Eukarya, Bacteria and Archaea, respectively. Median *f* values of these FSFs were considerably higher than those of corresponding cellular FSFs in Eukarya (0.978 vs. 0.416), Bacteria (0.8826 vs. 0.329), and Archaea (0.742 vs. 0.514) (Figure 5). This bias is remarkable in the case of Eukarya where nearly all (98%) the proteomes were enriched with viral FSFs (Figure 5). Archaeal and bacterial proteomes were also enriched with viral FSFs but at lower levels. Remarkably, patterns of enrichment follow patterns of reductive evolution in the superkingdoms (i.e., Archaea < Bacteria < Eukarya). The popularity and abundance of viral FSFs in cellular proteomes suggests that viruses have been a very active and crucial factor in mediating domain transfer between cellular species and enhancing biodiversity. These domains are present in a remarkably diverse array of cellular hosts, ranging from small microbes to complex vertebrates, providing further support to the ancient and primordial nature of viruses [16] and highlighting their crucial contribution to the biosphere [47]. 

### Functional makeup of viral proteomes

**Figure 6:**
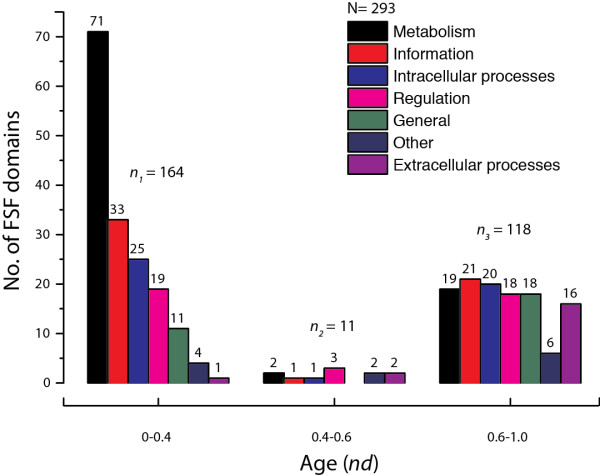
**Functional distribution of viral FSFs in major functional categories.** Histogram comparing the number of viral FSFs corresponding to major functional categories plotted against *nd*. The distribution of functions that appeared early and late is significantly biased. Numbers on top of individual bars indicate total number of FSFs corresponding to each functional category.

**Figure 7:**
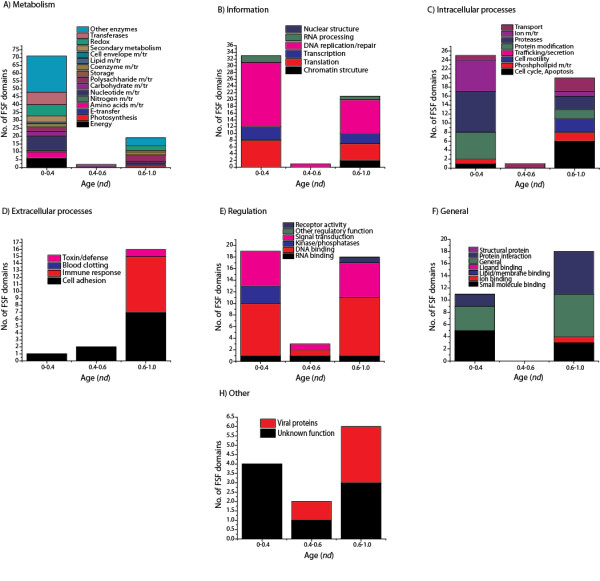
**Functional distribution of viral FSFs in minor functional categories.** Histograms comparing the number of viral FSFs corresponding to each of the minor categories within each major functional category.

We studied the molecular functions of 293 (out of 304) FSFs in viral proteomes using the functional annotation scheme described by Vogel and Chothia [59-61]. For the rest of the 11 FSFs, functional annotations were not available. When plotted against time (*nd*), we note that a majority (*n*_*1*_ = 164) of the viral FSFs either appeared very early (*nd* < 0.4) or very late (*n*_*3*_ = 118) (0.6 < *nd* < 1.0) (Figure 6), supporting timelines of Figure 1C. For the ancient FSFs (*nd* < 0.4), we note that most of the viral FSFs perform metabolic functions, followed by informational FSFs, *Intracellular processes*, *Regulation*, *General*, *Other* and *Extracellular processes*, in that order. This order matches the functional distribution described previously for the cellular superkingdoms [27]. A significant drop in the number of FSFs/functions is seen in the *nd* range 0.4-0.6 (*n*_*2*_ = 11) which is the period marked by massive gene loss in both viruses and cellular organisms. In contrast, a relatively even distribution of functions is seen in the *nd* range 0.6-1.0 which is the period marked by superkingdom diversification and genome expansion in Eukarya [28]. The specific functions acquired by viruses during this late period include those related to *Extracellular processes* (toxin/defense, immune response, cell adhesion), *General* (protein interaction, general, ion binding, small molecule binding), and *Other* (viral proteins and proteins with unknown functions) (Figure 7). We hypothesize that viruses acquired these functions in order to adapt to the parasitic lifestyle after suffering massive gene loss between *nd* 0.4-0.6. This is also evident by the appearance of superkingdom specific taxonomic groups (AV, BV, and EV) after the appearance of respective superkingdoms in Figure 1D. In contrast, the number of FSFs corresponding to *Metabolism*, *Information*, *Intracellular processes*, and *Regulation* is lower compared to *nd* < 0.4. The significant differences in the distribution of molecular functions for very early (*nd* < 0.6) and late periods (*nd* > 0.6) of the evolutionary timeline suggests that viruses started very much like cells (possibly as integral components of cells), experienced massive amounts of genome reduction and finally acquired specific structures and functions needed for a parasitic lifestyle in an expanding cellular world. 

### Effect of HGT

Domains defined at the FSF level are evolutionarily more conserved than domain sequences [23,29] and the evolutionary impact of HGT is limited at such levels of structural organization [31,32,34]. However, the HGT-derived domain structures are expected to be overrepresented in proteomes [41,67] and this significant enrichment of FSFs in viruses (in the ABEV, BEV, AEV, ABV, AV, BV, and EV taxonomic groups) is taken as an indication that viruses have acquired FSFs from their cellular hosts via HGT. We calculated the probability of enrichment of a particular taxonomic group using the hypergeometric distribution and found that only the ABEV FSF group was significantly overrepresented (*P* < 0.05). All the other taxonomic groups were significantly underrepresented (*P* < 0.05) with the exception of the AEV FSF group, which was overrepresented at statistically non-significant levels (*P* = 0.29, Table 4:Sampled viruses). Because HGT is thought to have played an important role in the evolution of prokaryotes, especially bacteria [68], we also evaluated the enrichment of FSFs in bacterial taxonomic groups (ABEV, BEV, ABV, ABE, BE, AB, and BV) as control to the enrichment test on viruses. We found that all the bacterial taxonomic groups were significantly overrepresented (*P* < 0.05) (as expected) except for ABEV and BV groups (Table 4:Bacteria), supporting high levels of HGT in Bacteria [68]. The significant underrepresentation of viral taxonomic groups indicates that FSFs encoded by giant viruses were not transferred laterally from their cellular hosts, though they can still contribute innovations to the structural make up of cells [28]. 

**Table 4:** Statistical test for the enrichment of FSFs in taxonomic groups using hypergeometric distribution for sampled viruses and bacteria

**Comparison**	***k***	***n***	***M***	***N***	***P-*value**	**Comment**
Sampled viruses						
ABEV_ABE	229	304	557	1739	9.80E-67	**Overrepresented**
BEV_BE	33	304	291	1739	0.0012	Underrepresented
BV_B	1	304	163	1739	1.80E-13	Underrepresented
BEV_BE	33	304	291	1739	0.0012	Underrepresented
ABV_AB	2	304	36	1739	0.035	Underrepresented
AV_A	1	304	22	1739	0.079	Underrepresented
AEV_AE	7	304	31	1739	0.29	Overrepresented
EV_E	25	304	335	1739	5.50E-09	Underrepresented
Bacteria						
ABEV_ABE	229	1312	557	1739	3.1E-111	Under-represented
BEV_EV	33	1312	25	1739	<0.0001	**Over-represented**
ABV_AV	2	1312	1	1739	<0.0001	**Over-represented**
ABE_AE	557	1312	31	1739	<0.0001	**Over-represented**
BE_E	297	1312	335	1739	9.9E-9	**Over-represented**
AB_A	36	1312	22	1739	<0.0001	**Over-represented**
BV_V	1	1312	6	1739	0.0044	Under-represented

Significantly over-represented comparisons are highlighted in boldface.

*k*, number of FSFs in viral taxonomic group; *n*, total number of FSFs in viral supergroup; *M*, number of FSFs cellular taxonomic group; *N*, total number of FSFs studied.

In order to evaluate the relative contributions of vertical and horizontal processes of inheritance in proteome evolution, we calculated the retention index (*r*_*i*_) for each phylogenetic character (*r*_*i*_ for each FSF) used to build the uToL as a relative measure of homoplasy (conflict in how data matches the reconstructed tree), and placed this information along the timelines generated from the trees of FSFs. *r*_*i*_ portrays processes other than vertical inheritance (i.e., convergent evolution and HGT) of characters (FSFs) on a relative scale of 0–1. Higher *r*_*i*_ values indicate a better fit of characters (FSFs) to the phylogeny and support vertical inheritance. We compared the *r*_*i*_ distribution of viral FSFs with the cellular FSFs and found that both groups of FSFs distribute similarly (Additional file 4: Figure S2). On average, however, *r*_*i*_ values were higher in viral characters (FSFs) (*r*_*i*_ = 0.79 for viral FSFs versus *r*_*i*_ = 0.71, *P* < 0.001), likely due to a significant drop of *r*_*i*_ values of FSFs of intermediate age (*nd* = 0.4-0.6), suggesting viral FSF characters fit better the uToL than cellular FSFs. Because viral and cellular FSFs distribute similarly in the timeline (Additional file 4: Figure S2), we conclude against intuition that the viral FSFs are not the subject of increased HGT levels relative to cellular FSFs.

## Discussion

In our analyses, we used the structural information of hundreds of proteomes to reconstruct the phylogenomic history of protein domains and organisms. We included viruses with medium-to-very-large proteomes and compared them to the cellular organisms. To our knowledge, this is the first exercise that makes extensive use of molecular data to study the evolution of viruses on a scale that is comparable to the cellular organisms. Additionally, we explored the functional annotations of protein domains (grouped into FSFs) and studied the proteomic make up of viruses. While structural information proved useful for the reconstruction of phylogenies, functional assignments described the nature of viral proteomes. This composite exercise of linking structure and function proved highly useful and yielded significant insights into the structure and evolution of organisms.

Our data provides evidence for high levels of genome reduction in giant viruses. This can be illustrated with the evolution of the most important brokers of translational specificity, the aaRS enzymes [64]. The existence of aaRSs in mimiviruses and megaviruses was recently given as evidence that these viruses evolved from an ancestral virus or cell harboring an entire aaRS set by reductive evolution [3]. The reduction hypothesis is more likely than the acquisition of mimiviral aaRS from its cellular host (i.e., amoeba) by HGT [18]. The probability of seven independent HGT events responsible for the seven aaRS enzymes of megavirus is unlikely [3] and has been toned down in several studies [9,19,69]. Abergel et al. [70] tested the activity of two of the mimiviral aaRS (MetRS and TyrRS) and found they were functionally active in infected amoebas. Because HGT-derived genes are expected to acquire different functions, ‘true’ activity of mimiviral enzymes favors the hypothesis of genome reduction in the mimiviral ancestor [70]. Similarly, sequence-based phylogenies did not suggest acquisition of aaRSs present in giant viruses via HGT either, since mimiviral sequences did not group with those of their eukaryotic host [3,70]. Our analysis of the distribution and evolution of aaRS domains is also incompatible with the HGT scenario. The wide distribution of catalytic, editing, trans-editing, anticodon binding and accesory domains of aaRS enzymes in cells constrasts with their limited presence or complete absence in mimiviruses, and their complete absence in the rest of dsDNA viruses we sampled (Figure 2). Even the average number of catalytic domains per proteome that are present in organisms of the cellular superkingdoms is highly reduced in mimiviruses, suggesting the specificity of aaRS enzymes needs to be supplemented with that of the amoebal host. The absence of crucial editing, anticodon-binding and accessory domains in viruses suggest forces of genome reduction that are similar to organisms exhibiting parasitic lifestyles (e.g., *Gillardia theta*) [27,28]. For example, the ValRS/IleRS/LeuRS editing domain that is ubiquitous in cellular proteomes and appears earlier than the anticodon binding domains is notoriously absent. Unless mimiviruses favor a statistical proteome, as those obtained by mutation of this editing domain [71], they must supplement their rudimentary aaRS repertoire with hijacked eukaryotic proteins. Especially relevant are the N-terminal d.67.2.1 additional domain of ArgRS, the C-terminal d.66.1.4 domain of TyrRS, and the b.40.4.4 Myf domain of MetRS and TyrRS, since these aaRS enzymes were identified in mimiviruses. These domains should have transferred from cellular superkingdoms together with core functional domains by HGT to enhance their catalytic activities. Their absence or extreme substructural reduction suggests again not only reductive evolution but also poor catalytic specificity of viral aaRSs and poor contribution, if any, to the many functions of aaRSs that are beyond translation [64]. In light of phylogenomic results, a more parsimonious explanation for the evolution of viral proteomes is their tendencies for genome reduction, anticipated in [28] and [38]. Thus, mimiviruses (and megaviruses) should be viewed as the least reduced forms of an ancestral virus or cell that either coevolved or predated the cellular ancestors.

The uToLs reconstructed from the total set of FSF domains (Figure 3A) and from only the set of universal FSFs (Addiftioanl file, Figure S1) showed that the cohesive viral supergroup exhibited very little diversity (shorter branches) compared to the diversity of cellular proteomes, which expands in the lineages of superkingdoms. We explain the lack of diversity in viral proteomes by their prolonged reductive evolutionary history and the fact that the set of FSFs that distinguish viruses are unevenly distributed in the virosphere (e.g., translation related enzymes are only present in mimiviruses and megaviruses). In contrast, longer branches in cellular superkingdoms are an indication of biological processes shaping the proteomes of higher organisms (i.e., accumulation of protein domains). We assume in our phylogenetic model that the ancestral proteome had a simpler organization (i.e., we treat FSF absence as the ancestral character state; see Methods) and hence resulted in a topology in which viruses appeared to be the most ancient supergroup. However and as explained above, the simpler organization of viral proteomes is a consequence of genome reduction and does not necessarily invalidate the ancestrality of viruses. Modern day viruses are recognized by smaller gene repertoires. It is possible they are also the reduced forms of an ancient viral lineage that coexisted with LUCA (very much like the dsDNA viruses we studied). It is less likely however that their initial origin was polyphyletic and driven by HGT from cells. The reconstruction of the ancestral viral lineage and testing the origins of all viruses seems possible with increased sampling of the virosphere and discovery of more giant viruses such as mimiviruses and megaviruses. The limited sampling of our study, mostly confined to viruses of the NCDLV group, and the existence of other giant viruses with even larger proteome repertoires of FSFs [3], does not affect the fundamental conclusion that the origin of viruses, at least giant viruses, was primordial and their evolution was shaped by reductive processes. A phylogenomic analysis involving all viral genomes that are known will help dissect primary and secondary adaptations shaping viral diversity along the entire evolutionary timeline (Nasir et al., ms. in preparation).

## Conclusions

We show that viruses with medium-to-very large proteomes harbor a significant number of FSFs and suggest that they have evolved via massive reductive evolutionary processes that are not so uncommon for small bacteria with small genomes and similar parasitic lifestyles. In addition, these viruses appear as a distinct supergroup on a uToL along with the three cellular superkingdoms. We propose that the viruses we have analyzed coexisted with primordial cells. These primordial entities could have been integral components of the common ancestor of life. We finally highlight the crucial contribution of the virosphere to biodiversity by mediating HGT between cellular species.

## Abbreviations

FSF: Fold superfamily; FF: Fold family; F: Fold; FL: Free living; P: Parasitic; OP: Obligate parasitic; HGT: Horizontal gene transfer; LUCA: Last universal common ancestor; SCOP: Structural Classification of Proteins; PDB: Protein data bank; NCLDV: Nucleo-cytoplasmic large DNA viruses; aaRS: Aminoacyl-tRNA synthetases; ToD: Tree of domains; ToP: Tree of proteomes; uToL: Universal Tree of Life.

## Competing interests

The authors declare no competing interests.

## Authors’ contributions

All authors designed experiments, performed experiments and read and approved the final manuscript.

## Supplementary Material

Additional file 1: **Table S1.** Statistics on the assignment of FSFs in supergroups. Click here for file

Additional file 2 **Table S2.** List of domain FFs that make structural components of aaRS enzymes. Click here for file

Additional file 3 **Figure S1.** Universal tree of life (uToL) reconstructed from the set of universal FSFs and corresponding network tree representation. A. One optimal most parsimonious phylogenomic tree describing the evolution of 200 proteomes equally sampled from supergroups (50 each from Archaea, Bacteria, and Eukarya and viruses) generated using the census of abundance of 229 FSFs in the ABEV taxonomic group (229 parsimony informative characters; 16,642 steps; CI = 0.1302; RI = 0.8233; *g*_1_ = −0.399). Terminal leaves of viruses, Archaea, Bacteria, and Eukarya were labeled in red, blue, green, and black respectively. Numbers on the branches indicate bootstrap values. B. Network tree generated from the presence/absence matrix of 229 FSFs (228 non-constant character sites) in 200 sampled proteomes. Nodes were represented by rectangles and labeled as in A. Numbers on the major splits indicate bootstrap values. CI, consistency index; RI, retention index; g_1_, tree skewness. Click here for file

Additional file 4 **Figure S2.** Retention index (*r*_*i*_) of each FSF plotted against relative age (*nd*). Viral FSFs are colored red as above and cellular FSFs are represented in blue. Both groups of FSFs follow an identical distribution and generally the viral FSFs are distributed with higher *r*_*i*_ values supporting a better fit of viral characters to the phylogeny. Click here for file
