# Supplementary material for: Giant viruses coexisted with the cellular ancestors and represent a distinct supergroup along with superkingdoms Archaea, Bacteria and Eukarya
Source: BMC Evol Biol. 2012 Aug 24;12:156. doi: 10.1186/1471-2148-12-156 (PMC3570343; doi:10.1186/1471-2148-12-156)
Supplement: Additional file 1: — Table S1. Statistics on the assignment of FSFs in supergroups. [file 1471-2148-12-156-S1.doc]

**Table S1 Statistics on the assignment of FSFs in supergroups.**

| **Supergroup** | ***N*** | **Mean proteomic**  **coverage** | **Median proteomic**  **coverage** | **Min** | **Max** |
| --- | --- | --- | --- | --- | --- |
| Viruses^1^ | 56 | 34.3% | 34% | 5%^a^ | 59%^b^ |
| Archaea | 70 | 61.4% | 61% | 52%^c^ | 71%^d^ |
| Bacteria | 652 | 65.5% | 66% | 44%^e^ | 88%^f^ |
| Eukarya | 259 | 55.2% | 55% | 22%^g^ | 80%^h^ |

^1^Virus families included: Adenoviridae, Ascoviridae, Asfarviridae, Corticoviridae, Iridoviridae, Mimiviridae, Phycodnaviridae, Poxviridae, Rudiviridae, and Tectiviridae.

^a^*Pseudoalteromonas* phage PM2

^b^*Acanthamoeba* *polyphaga* mimivirus)

^c^*Pyrobaculum aerophilum*

^d^*Thermoplasma acidophilum*)

^e^*Microcystis aeruginosa*

^f^*Cand.Blochmannia floridanus*

^g^*Plasmodium chabaudi*

^g^*Takifugu rubripes*)

*N*, total number of proteomes studied; Mean proteomic coverage, average number of proteins with assignments / by total number of proteins; Median proteomic coverage, median number of proteins with assignments / by total number of proteins *Min*, minimum proteomic coverage in a supergroup; *Max*, maximum proteomic coverage in a supergroup;
