# Supplementary material for: Giant viruses coexisted with the cellular ancestors and represent a distinct supergroup along with superkingdoms Archaea, Bacteria and Eukarya
Source: BMC Evol Biol. 2012 Aug 24;12:156. doi: 10.1186/1471-2148-12-156 (PMC3570343; doi:10.1186/1471-2148-12-156)
Supplement: Additional file 2 — Table S2. List of domain FFs that make structural components of aaRS enzymes. [file 1471-2148-12-156-S2.doc]

**Table S2 List of domain FFs that make structural components of aaRS enzymes.**1

| **No.** | **SCOP identifier** | **FF *ccs*** | **FF name** | **Domain•** | **Taxonomic coverage** |
| --- | --- | --- | --- | --- | --- |
| 1 | 64587 | d.68.5.1 | C-terminal domain of ProRS | Accessory | AB |
| 2 | 50277 | b.40.4.4 | Myf domain | Accessory | ABE |
| 3 | 55262 | d.74.4.1 | GAD domain | Accessory | ABE |
| 4 | 46593 | a.2.7.2 | Phenylalanyl-tRNA synthetase (PheRS) | Accessory | B |
| 5 | 81635 | a.2.7.3 | Valyl-tRNA synthetase (ValRS) C-terminal domain | Accessory | B |
| 6 | 46956 | a.6.1.1 | Domains B1 and B5 of PheRS-beta, PheT | Accessory | B |
| 7 | 81270 | d.15.10.1 | TGS domain | Accessory | B |
| 8 | 103046 | d.66.1.6 | YbcJ-like | Accessory | B |
| 9 | 64294 | d.185.1.2 | Autoinducer-2 production protein LuxS | Accessory | B |
| 10 | 55191 | d.67.2.1 | Arginyl-tRNA synthetase (ArgRS), N-terminal 'additional' domain | Accessory | BE |
| 11 | 74759 | a.97.1.2 | C-terminal domain of class I lysyl-tRNA synthetase | AC-binding | AB |
| 12 | 101354 | a.203.1.1 | Putative anticodon-binding domain of alanyl-tRNA synthetase (AlaRS) | AC-binding | ABE |
| 13 | 50250 | b.40.4.1 | Anticodon-binding domain | AC-binding | ABE |
| 14 | 47324 | a.27.1.1 | Anticodon-binding domain of a subclass of class I aminoacyl-tRNA synthetases | AC-binding | ABEV |
| 15 | 52955 | c.51.1.1 | Anticodon-binding domain of Class II aaRS | AC-binding | ABEV |
| 16 | 46590 | a.2.7.1 | Seryl-tRNA synthetase (SerRS) | AC-binding | B |
| 17 | 48164 | a.97.1.1 | C-terminal domain of glutamyl-tRNA synthetase (GluRS) | AC-binding | B |
| 18 | 54992 | d.58.13.1 | Anticodon-binding domain of PheRS | AC-binding | B |
| 19 | 75465 | d.66.1.4 | Tyrosyl-tRNA synthetase (TyrRS), C-terminal domain | AC-binding | B |
| 20 | 50719 | b.53.1.2 | Gln-tRNA synthetase (GlnRS), C-terminal (anticodon-binding) domain | AC-binding | BE |
| 21 | 47068 | a.16.1.3 | a tRNA synthase domain | AC-binding | E |
| 22 | 55682 | d.104.1.1 | Class II aminoacyl-tRNA synthetase (aaRS)-like, catalytic domain | Catalytic | ABE |
| 23 | 52375 | c.26.1.1 | Class I aminoacyl-tRNA synthetases (RS), catalytic domain | Catalytic | ABEV |
| 24 | 103051 | d.67.1.2 | AlaX-like | Editing | A |
| 25 | 50678 | b.51.1.1 | ValRS/IleRS/LeuRS editing domain | Editing | ABE |
| 26 | 55187 | d.67.1.1 | Threonyl-tRNA synthetase (ThrRS), second 'additional' domain | Editing | ABE |
| 27 | 55827 | d.116.1.1 | YbaK/ProRS associated domain | Editing | ABE |
| 28 | 56038 | b.153.1.1 | B3/B4 domain of PheRS, PheT | Editing | BE |

^1^Abbreviations: *ccs,* concise classification string; AC, anticodon; A, Archaea; B, Bacteria; E, Eukarya; V, viruses.
