# Supplementary material for: Giant viruses coexisted with the cellular ancestors and represent a distinct supergroup along with superkingdoms Archaea, Bacteria and Eukarya
Source: BMC Evol Biol. 2012 Aug 24;12:156. doi: 10.1186/1471-2148-12-156 (PMC3570343; doi:10.1186/1471-2148-12-156)
Supplement: Additional file 3 — Figure S1. Universal tree of life (uToL) reconstructed from the set of universal FSFs and corresponding network tree representation. A. One optimal most parsimonious phylogenomic tree describing the evolution of 200 proteomes equally sampled from supergroups (50 each from Archaea, Bacteria, and Eukarya and viruses) generated using the census of abundance of 229 FSFs in the ABEV taxonomic group (229 parsimony informative characters; 16,642 steps; CI = 0.1302; RI = 0.8233; g1 = −0.399). Terminal leaves of viruses, Archaea, Bacteria, and Eukarya were labeled in red, blue, green, and black respectively. Numbers on the branches indicate bootstrap values. B. Network tree generated from the presence/absence matrix of 229 FSFs (228 non-constant character sites) in 200 sampled proteomes. Nodes were represented by rectangles and labeled as in A. Numbers on the major splits indicate bootstrap values. CI, consistency index; RI, retention index; g1, tree skewness. [file 1471-2148-12-156-S3.doc]

**
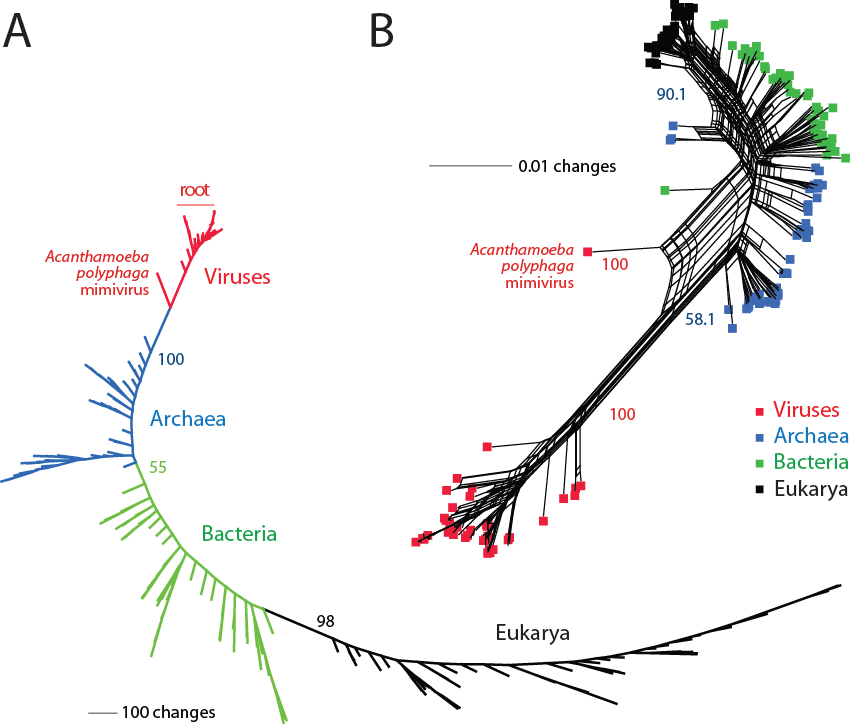
**

**Figure S1 Universal tree of life (uToL) reconstructed from the set of universal FSFs and corresponding network tree representation.** A. One optimal (*P*  < 0.01) most parsimonious phylogenomic tree describing the evolution of 200 proteomes equally sampled from supergroups (50 each from Archaea, Bacteria, and Eukarya and viruses) generated using the census of abundance of 229 FSFs in the ABEV taxonomic group (229 parsimony informative characters; 16,642 steps; CI = 0.1302; RI = 0.8233; *g*_1_ = –0.399). Terminal leaves of viruses, Archaea, Bacteria, and Eukarya were labeled in red, blue, black and green, respectively. Numbers on the branches indicate bootstrap values. B. Network tree generated from the presence/absence matrix of 229 FSFs (228 non-constant character sites) in 200 sampled proteomes. Nodes were represented by rectangles and labeled as in A. Numbers on the major splits indicate bootstrap values. CI, consistency index; RI, retention index; g_1_, tree skewness
