# Supplementary material for: Giant viruses coexisted with the cellular ancestors and represent a distinct supergroup along with superkingdoms Archaea, Bacteria and Eukarya
Source: BMC Evol Biol. 2012 Aug 24;12:156. doi: 10.1186/1471-2148-12-156 (PMC3570343; doi:10.1186/1471-2148-12-156)
Supplement: Additional file 4 — Figure S2. Retention index (ri) of each FSF plotted against relative age (nd). Viral FSFs are colored red as above and cellular FSFs are represented in blue. Both groups of FSFs follow an identical distribution and generally the viral FSFs are distributed with higher ri values supporting a better fit of viral characters to the phylogeny. [file 1471-2148-12-156-S4.doc]

**
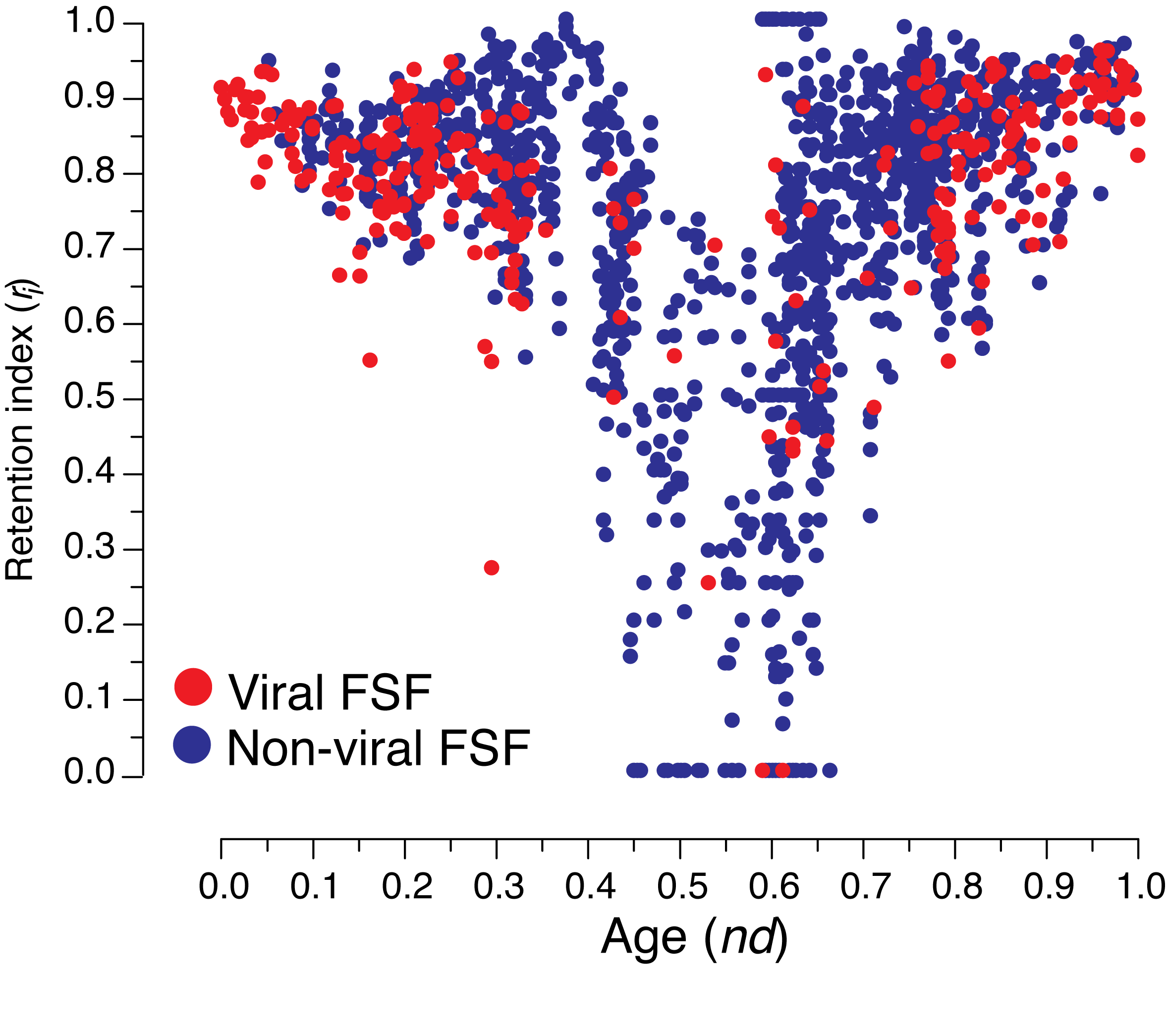
**

**Figure S2 Retention index (*r_i_*) of each FSF plotted against relative age (*nd*).** Viral FSFs are colored red as above and cellular FSFs are represented in blue. Both groups of FSFs follow an identical distribution and generally the viral FSFs are distributed with higher *r_i_* values supporting a better fit of viral characters to the phylogeny.
